# Supplementary material for: YouTube as a source of information on ectopic pregnancy: A qualitative and quantitative analysis
Source: PLoS One. 2023 Jul 12;18(7):e0287603. doi: 10.1371/journal.pone.0287603 (PMC10337927; doi:10.1371/journal.pone.0287603)
Supplement: Attachment — Submitted filename: Response to Reviewers.docx [file pone.0287603.s003.docx]

^1^BULLE Camille

Department of Obstetrics and Gynecology

Besancon University Medical Centre

3 Alexander Fleming Boulevard

25000 Besançon, France

bullecam@gmail.com

Angelica Espinosa Miranda, M.D., Ph.D.
 Academic Editor
 PLOS ONE

May 18, 2023,

**Object**: My response to the comments from the reviewers

**Manuscript ID:** PONE-D-23-11462R1

**Title:** YouTube as a source of information on ectopic pregnancy: a qualitative and quantitative analysis

Dear Angelica Espinosa Miranda,

We thank you and the reviewers for your time in reviewing our article. I carried out the corrections as suggested by reviewers. You will find below my response to the comments.

Reviewer 1.

**Introduction

The authors need to improve the introduction of the article a lot.

It is necessary to better contextualize the problem and present epidemiological data on ectopic pregnancy. It is not enough simply to state that this can be a fatal pregnancy if left undetected, and that this is mainly a problem in developing countries. I consider it fundamental that the authors present data on ectopic pregnancy in some countries, this will qualify the statements made. For example, show data on deaths and ectopic pregnancies in developed and developing countries.**

All these suggestions have been considered and appear highlighted in the text (lines 82 – 85).

**In the introduction, the authors should make clear the objective of the article and present the research questions.**

All these suggestions have been considered and appear highlighted in the text (lines 94– 95 and 100)

**Methodology

The methodology is well described, however, I recommend that the authors place in a supplementary file a list of all the videos that were selected for the study. This is important to ensure method replication as well as research transparency.**

The supplementary file has been uploaded as “YouTube videos link”.
 **Authors should create a subsection to better explain the DISCERN tool.**

A subsection has been created. (line 155).

 **Related works

Authors must include a related work section. It is important to know if there are other works and what these works are doing different from the one presented by the authors.

The related work section should be a section where the authors will deepen the discussion on topics based on the works found.

Authors can use more recent works, between 2019 to 2023 and make a critical analysis of the works. The authors state in the discussion that this article was the first to evaluate data from YouTube videos on ectopic pregnancy. However, to better qualify this statement, the authors must present it in this section of related works**.

After a long review of the literature, there are very few studies about YouTube information, and none about YouTube videos and ectopic pregnancy. The methodology applied in this article is the same as in the other articles dealing with the quality of YouTube videos on other topics. The only differences are explained in the discussion section.

**Discussions

The authors make the following statement in the discussion: "YouTube’s algorithm might skew the order of the top 30 videos, as the videos order changes following the viewing of previous videos. This constitutes a selection bias."
I agree that the display of videos changes depending on the YouTube algorithm. However, why would that be a problem? The authors should explain this part better - only the issue of bias in the selection of videos is not enough.**

The suggestion has been considered. ( line 342)

**Finally, the authors must include in the discussion a list of recommendations so that a video has quality and at the same time can dialogue (be understandable) with the population (accessible content). It is necessary to deepen this point a little in the discussion and talk about non-formal education in health. The authors talk about this in the conclusions, but in a very superficial way**.

The suggestions have been considered. (line 328)

**Reviewer 2**

**Dear Authors
I begin by congratulating the authors for the work presented.
Great impact on women's health, mental health and public health in general.
Some questions:
- in table 2 the number of likes is one of the most complex variables. Very asymmetrical;**

The table has been modified.

**- in table 3, I suggest you include the year of production of the video (the content may be less or more updated);**

The table has been modified.

**Conclusions can be improved. As a suggestion the involvement of the university and health professionals in the execution of these videos.
They must include** **the sustainable development goals (Agenda 2023) to reinforce the pertinence of the theme and the objectives of this article.**

The suggestions have been considered. (line 317)

Now that the corrections are made, I hope you will find the manuscript acceptable for publication in PLOS ONE Journal. Please feel free to contact us with any questions or concerns.

Thank you for your consideration.

Sincerely,

Camille BULLE
